# Role of Polyetheretherketone in Prosthodontics: A Literature Review

**DOI:** 10.7759/cureus.60552

**Published:** 2024-05-18

**Authors:** Fazail Ahmad, Sharayu Nimonkar, Vikram Belkhode, Pranali Nimonkar

**Affiliations:** 1 Prosthodontics, Sharad Pawar Dental College, Datta Meghe Institute of Higher Education and Research, Wardha, IND; 2 Trauma Care Centre, Government Medical College and Hospital, Nagpur, IND

**Keywords:** polymers, esthetic, titanium, polyetheretherketone, implant prostheses

## Abstract

Implant prostheses and other fixed and removable metal prostheses have led to an increase in demand for the development of new and efficient materials such as high-performance polymers polyetheretherketone (PEEK) over titanium and other metals because of their further complications in the human body. PEEK is a polymer that is nontoxic and has a modulus of elasticity that is comparable to that of human bone. PEEK implants provide benefits over metal implants, such as reducing the stress shielding effect, simple processing, and color resemblance to natural teeth. And it is a fantastic alternative to titanium for dental and orthopedic implants. The current review is undertaken to understand the properties of this PEEK material to weigh its benefits and drawbacks for potential use in dental implants and other prostheses.

## Introduction and background

Polyether ether ketone is a semicrystalline structural thermoplastic with exceptional strength characteristics and great resistance to chemicals, wear, and creep. Polyether ketone (PEEK), polyether ether ketone ketone (PEEKK), polyether ketone ketone (PEKK), and other polymers are contained in a much broader class of (PAEK) polymers. Among the variations, PEEK is the most well-known polymer [1].

Polyetheretherketone (PEEK) is a linear, aromatic, semi-crystalline thermoplastic. Extremely durable polymer has been used to create endocrowns, resin-bonded types of fixed prostheses, removable types of dental prostheses, implant-supported types of fixed prostheses, implant-retained types of overdentures, and metal-free fixed dental prostheses [2].

Dental implants have become the most trending and successful treatment for patients with missing teeth, they not only improve the esthetics of the patient but also enhance the quality of life of a patient [3]. The preferred material for dental implants is titanium, which was introduced by Branemark towards the end of 1960 [4]. Although titanium and titanium alloy implants have further demonstrated that their usage can be associated with a variety of issues. A few common problems related to titanium implants are that titanium has a huge difference in Young’s modulus of elasticity when compared to human bone. Titanium’s modulus of elasticity is (110 GPa [Grade point average]) whereas for bone it is (14 GPa). This significant disparity frequently causes implant failure owing to inadequate stress shielding, bone resorption, and implant fracture [5]. Additionally, titanium lacks light transmission which leads to aesthetic issues. If the biotype mucosa around a titanium implant is thin or has receded, this may cause the peri-implant soft tissue to shimmer darkly. This may be a concern, particularly if there is a prominent smile line. Furthermore, a growing number of patients are preferring dental reconstructions made entirely of materials devoid of metal [6]. To overcome all these issues polyether ether ketone (PEEK) is the best alternative. PEEK is synthesized at 300 degrees Celsius (C) and a reaction occurs between “4,4-difluoro benzophenone” and “sodium salt of hydroquinone” in a polar solvent. Melting of PEEK takes place at 335 C and has a color of tooth synthetic polymeric material which is frequently used as a biomaterial in orthopedics for long [7]. It is biocompatible, has strong mechanical properties, and is chemically resistant. PEEK is utilized as the superstructure, abutment, and fixture of implants. Young's modulus of elasticity of a PEEK material is 3.6 GPa in its unalloyed form, 18 GPa or more in alloyed form that is carbon-reinforced PEEK (CRF-PEEK), and 12 GPa in glass fiber-reinforced PEEK (GFR-PEEK). PEEK displays less stress shielding than Titanium (Ti) because its Young's modulus of elasticity is close to that of the cortical bone (14GPA) [2]. PEEK has relatively fewer inherent osteoconductive characteristics, in contrast to titanium. As a result, the bioactivity of PEEK implants has been the subject of substantial research [8].

PEEK has been suggested to be made more bioactive using a variety of methods, such as layering it with synthetic “osteoconductive hydroxyl apatite”, roughening up its surface, chemically modifying it, and adding bioactive particles [9]. Henceforth PEEK can be a great future material for clinical dentistry in prosthodontics such as implant abutments, removable and fixed denture, their components, and fixed crowns, even if it can be used as esthetic wire in orthodontics. PEEK orthodontic wires can produce stronger orthodontic forces than other polymers like “polyether sulfone (PES) and polyvinylidene difluoride (PVDF)” and also have specimens similar to metallic wires like “cobalt-chromium (Co-Cr), titanium-molybdenum (Ti-Mo), and nickel-titanium (Ni-Ti)”. PEEK can be a promising material for clinical dentistry. This review's objective is to provide an overview of the findings from research on the PEEK material used in prosthetic applications.

## Review

PEEK plastic is a widely sought-after engineering material because of its exceptional combination of workability, resistance, and strength. PEEK plastic is a semicrystalline engineering thermoplastic belonging to the polyaryletherketone (PAEK) family, which is known for its strength, stability, and ability to withstand high temperatures in a variety of challenging conditions [3]. Because of the stiff aromatic units used in its manufacturing, it has a high melting point of 343 °C/649 °F. PEEK is available in a variety of grades that are either extremely pure PEEK or have other technical polymers, carbon fiber, or glass reinforced in it. Twenty percent of the publications discuss PEEK's properties, demonstrating its superior mechanical qualities compared to current dental metals. According to documented data, PEEK's physical characteristics include an elastic modulus of 3.6 GPa. By adding carbon fibers, this modulus can be increased to 18 GPa, or nearly 15 GPa, which is comparable to cortical bone [2,5,8]. Due to its radiolucency, PEEK exhibits less artifacts in magnetic resonance imaging and has a flexural strength of 140-170 MPa, making it extremely rigid. The fact that PEEK is not about opposing natural teeth is yet another fantastic benefit [5]. The US FDA Drug & Device Master files attest to its biocompatibility and bio-stability. Due to its superior polishing qualities, low plaque affinity, non-metallic taste, and good wear resistance, Bio HPP, the modified form of PEEK, offers more advantages [9].

PEEK in implantology

“Dental implantology” is the most effective prosthesis in treating or replacing missing teeth. The first and most preferred material of choice in the field of dental implants is titanium. Due to the significant dissimilarity between the modulus of elasticity of bone (1-30 GPa) and titanium (110 GPa) during mastication, overloading of the jawbone will be a matter of concern, which can pose serious issues for the related implants. In this circumstance, PEEK can be an encouraging material of choice because of its aesthetics, physical, and mechanical properties along with its elastic modulus (3-4 GPa) in contrast to Ti. PEEK has a unique structure that optimizes how masticatory forces are distributed around the implant [10]. PEEK was further modified so that it could be used in implants. PEEK implants layered in “hydroxyapatite (HA)” are a superior material for curative devices that require to be grasped in position by bone. A “nanosized PEEK” that has been treated with “Hydroxyapatite”, assembles a 20 to 40 nm plenty thickness of “nanosized matter” with dimension, arrangement, and translucency the same as that of anthropoidal bone [11]. Sandblasted PEEK was discovered to be extremely well osseointegrated to its bone matrix and to have outstanding osteogenic characteristics and biocompatibility [12]. With a stronger shear connection between the bone and the implant, Titanium Dioxide (TiO2) coatings on PEEK implants helped to begin new bone formation. Thirty percent Carbon Fiber Reinforced (CFR-PEEK) provided elevated stress concentration near the implant neck and the adjacent bone, even though CFR-PEEK demonstrated an optimal interaction between bone and implant [13]. Research has demonstrated that fatigue strength is extremely strong and can serve as an implant's superstructure. PEEK can thus be utilized as the implant-retained prosthesis' superstructure [9]. Table 1 compares the mechanical and physical properties of PEEK, GFR-PEEK, and CFR-PEEK.

**Table 1:** The mechanical and physical properties PEEK: Polyether Ether Ketone, GFR: Glass Fiber-Reinforced, CFR: Carbon Fiber Reinforced, g: gram, cm: centimeter, GPa: Grade Point Average, MPa: MegaPascal, %: percentage

Mechanical and Physical Properties	PEEK	GFR-PEEK	CFR-PEEK
Specific gravity (g/cm^3^)	1.31	1.51	1.41
Young’s modulus (GPa)	3-4	12	18
Radiolucency (MPa)	140-170		
Tensile strength (MPa)	110	97	131
Tensile modulus of elasticity (GPa)	4.3	6.9	7.6
Tensile elongation (%)	40	2	5

“PEEK implants” in specific and implants layered with PEEK materials may have fewer results on stress protection. In bioactive PEEK the nanoparticles are combined with bioactive particles to create bioactive implants. It has been demonstrated that the hydroxyapatite particles, which is a bio-ceramic, have properties similar to that of bone, and induce bone formation surrounding implants [14]. PEEK is blended with certain micrometer-sized particles of hydroxyapatite to form Poly ether ether ketone Hydroxyapatite (PEEK-HAp) composites, which have enhanced mechanical properties that increase the bioactivity of the PEEK. The technique used for making PEEK-HAp composites is the melt blending technique [15]. However, there were few variations in certain studies, more such investigation and research are required to claim further use of PEEK in clinics. Surface modification of PEEK is to make PEEK more biologically active; there are different types of surface modifications present. In comparison to nanocomposite PEEK, surface modification makes changes only on the surface area and does not affect significantly less to the core. Until now four methods have been introduced to nano-modify the surface of PEEK implants to enhance its properties. They are spin-coating of PEEK implants, gas plasma nano etching of PEEK implants, “electron beam deposition” on PEEK implants, and plasma-ion immersion implantation on PEEK implants.

Spin coating has been done to cover implants with thinner coatings, because of the disadvantages of thick hydroxyapatite (HA) layering [14]. A thin layering of nano-HA that has been emersed in wetting agents such as organic and aqueous solvents of Calcium nitrate (Ca(NO3)2)” and “Orthophosphoric Acid (H3PO4)” is applied to the implants throughout spin-coating. Barkarmo et al. published the first study for spin-coated PEEK implants in which they stated that there was not much of a difference in the average taking away torque of spin-coated implant discs when compared with uncoated implants [16]. Another study done by Barkarmo et al. suggested greater removal of torque when compared to uncoated PEEK [17]. Another study by Johansson et al. suggested that by changing the design and adding a threaded cylindrical design of the implants, there was greater torque removal than the uncoated PEEK implants [11]. The results indicated that effective PEEK dental subgingival implants require both an adequate implant design and an appropriate bioactive covering [18].

Gas plasma etching in implants made up of PEEK can be nano-etched via exposure to “low-intensity plasma gases” such as ammonia, oxygen/argon, and water vapor. It has been proposed that PEEK treated with plasma adds a variety of functional groups to its surface, increasing its hydrophilicity [19]. The two main advantages of utilizing “plasma treatment” are the capacity to fabricate “nano-level roughness” on the implant surface and the shallow water-contact angle on the surface of PEEK. In fact, in vitro investigations have shown that plasma-etched PEEK implants accelerate the growth of human mesenchymal cells [20]. This is thought to occur as a result of the increased protein adsorption and hydrophilicity caused by nano roughness. Due to the lack of layering in implants with “plasma-etched”, there is no possibility of layering delamination.

The veneer characteristics of PEEK having plasma treatment have been shown to deteriorate with time. However, it has also been documented by Say et al. that pre-treating PEEK with a “pulsed Nd: YAG (Neodymium-doped yttrium aluminum garnet) laser” can extend the benefits and effects of plasma treatment [21]. Electron beam deposition is a non-volatile fragment that is broken down and deposited on a substrate using an electron beam deposition method. It has been demonstrated that an ultra-thin titanium layering applied to PEEK making use of electron beam deposition increases wettability and encourages cellular attachment. Anodizing transforms a titanium coating created by electron beam deposition on PEEK into a uniformly thick, smooth, and very nano-porous layer of titanium oxide that may be employed to transport bone morphogenetic protein (BMP) [22]. Numerous articles have shown in-vitro and in-vivo investigations demonstrate that BMP is a growth factor that is essential for the differentiation of stem cells into osteoblasts. In this, an immobilized growth factor on the implant's surface may speed up osseointegration surrounding it. Plasma-ion immersion implantation is a thin coating of various particles that can cover a substrate, putting it in a plasma of the particles. The plasma ions are then repeatedly accelerated by strong negative voltage pulses and implanted onto the substrate's surface. This procedure is known as plasma immersion ion implantation. The literature says that PEEK implants may provide some antibacterial effects against Escherichia coli and Staphylococcus aureus [23]. Using plasma immersion ion implantation, nano-TiO2 (Titanium Dioxide) particles may be deposited onto PEEK. Although it has also been demonstrated that diamond-like carbon coated on PEEK exhibits higher bioactivity in vitro, the consequences of the surface modification in vivo have not yet been examined [24]. A study by Mishra et al. concluded that with PEEK as the framework, the cumulative survival rate of prosthesis was 97.3%. PEEK and PEKK frameworks were reported to have prosthetic fractures and problems. No implant failure has been documented using PEKK or PEEK [18]. According to a study by Mahalakshmi et al., there were no appreciable variations in the three implant assemblies - carbon fiber-reinforced PEEK, titanium, and zirconia - and their respective bone stresses and deformations were comparable [20].

PEEK in fixed partial dentures

For fixed partial dentures (FPD), the main factors to take into account are stress distribution, fracture resistance, and fracture pattern (FPDs). PEEK has an elastic modulus of 3 to 4 GPa that is lower than that of zirconia and Co-Cr alloys (220 GPa) [25]. Due to this benefit, PEEK offers stress resistance for the abutment teeth when the occlusal forces are directed towards the pontic, preventing them from getting a fracture. Greater stress distribution was supplied by the connectors in the PEEK prosthesis than by any other components. PEEK may reduce the amount of stress that accumulates in FPDs since it has the lowest strength of any cement layer when measured through the cervical margin. The occlusal region of PEEK shows the greatest degree of strengthening. Rauch et al. claim that PEEK needs minimal manufacturing time and is lighter in weight than zirconia when comparing the clinical application of PEEK FPDs to zirconia. He also added that both materials are visually acceptable, despite zirconia having produced better-looking results than veneered PEEK [26]. When evaluated using the California dental assessment system and the modified Ryge Criteria, PEEK FPDs produce good clinical results [27]. Only 6% of PEEK FPDs failed due to de-bonding, and the remainder were maintained without fracture. Fifteen percent of the restorations displayed marginal discoloration, although marginal adaptation did not alter significantly over a year. Despite this, when inlay-retained FPDs made of PEEK and other resin-based materials were examined, PEEK demonstrated a superior capability for load bearing [28]. To assess the long-term usage of PEEK FPDs, more clinical trials are needed. Additionally, it was stated that cast-metal and glass-fiber posts might be replaced with milled PEEK intra-radicular posts. When combined with the right adhesive technique and surface treatment, PEEK posts outperformed metal and glass-fiber posts in terms of tensile bond strength, according to in vitro research. PEEK has been tested as a post-core intra-radicular material [27]. According to the studies, PEEK posts have a higher fracture resistance than other popular post and core materials, and employing PEEK also reduces the risk of root fracture [23].

PEEK in removable dental prostheses

The invention of the CAD-CAM (computer-aided design and manufacturing) mechanism has resulted in the development of several newer materials that can be accurately utilized to fabricate removable dental prostheses (RPD) [29]. RPD frameworks can also be produced using CAD-CAM procedures. According to the published clinical reports by Harb et al., PEEK frameworks can be used instead of conventional Co-Cr frameworks [30]. This is made feasible by combining the PEEK substructure with the bases made in acrylic resins and heat-cured acrylic resin denture teeth [31]. PEEK offers significant qualities, excellent biocompatibility, and low specific weight which makes it possible to create lighter, white hue, and metal-free RPDs by removing the esthetically poor metal claps, the danger of allergies, and metallic taste from traditional RDP metal frameworks. Other studies have shown milled PEEK can be used for making an obturator in maxillary arches, further feedback from patients was also exceptionally good for its retention comfort and esthetics [30]. PEEK's high degree of elasticity made it possible to minimize distal torque and stresses on the abutment teeth while it was functioning. PEEK frameworks cause the periodontal ligament to be under less stress than cobalt-chromium and Ti alloy, according to Chen et al.'s 3-D, finite element analysis, which justifies the above statement. PEEK RPDs may be suggested for individuals with severe periodontal problems [31]. With the use of CAD-CAM, RPD can be created utilizing a variety of techniques, such as directly milled PEEK blanks or 3-D printing of a framework made of resin and wax that is subsequently thermo-pressed using the traditional lost-wax/resin method. Further, more clinical investigations are required to understand the long-term use of PEEK for removable dental appliances.

PEEK in maxillofacial prostheses

Closing oronasal communication and returning it to function is the main objective of rehabilitation for patients with intra-oral maxillary or mandibular maxillofacial defects. Owing to PEEK's superior biocompatibility, the obturator framework was able to close the patency and provide a lightweight prosthesis for the massive maxillectomy defect. Furthermore, advised for occlusal splints made using CAD-CAM technology is the usage of PEEK. When PEEK occlusal splints were compared with other CAD-CAM materials like “vinyl acetate (EVA), polymethyl methacrylate (PMMA), polycarbonate (PC), and polyethylene terephthalate (PETG)”, an in vitro investigation revealed reduced volume loss and change in roughness. PEEK polymer possesses certain highly desirable qualities for any material intended for use in the fabrication of an ocular prosthesis. Weight is among the most crucial characteristics. Numerous methods are described in the literature to reduce the weight of the polymethyl methacrylate (PMMA) obturator prosthesis. Conversely, PEEK prostheses weigh far less than those constructed of conventional materials. Weightlessness, biocompatibility, and polish ability will soon make PEEK polymer the material of choice [22].

PEEK is effectively utilized in oral and maxillofacial surgery for surgical guides for implant insertion, maxillofacial bone repair, and occlusal splints. When compared to tiny titanium plates, some writers discovered that plates made up of PEEK plates did not appear to ensure the replacement and mechanical ethics of mandibular restoration [28]. Others, on the other hand, came to the conclusion that the CF/PEEK plate/screw system offers enhanced steady fixation than the resorbable organization and lessens the stress on the fixation system [19,21,23]. When treating displaced mandibular fractures, the 1 mm thick titanium plate may be replaced by the 2 mm thick CF/PEEK plate [28]. Because PEEK may be used to manage the volumetric geometry and internal connection of the tissue scaffold, it is frequently manufactured into three-dimensional porous scaffolds for the treatment of large bony defects in addition to its application in the fixing of mandibular fractures. Highly porous, bionic PEEK bone scaffolds were created using melt casting and salt-based pyrogenic (200-500 micron size) leaching techniques.

Changes in the PEEK powder's salt content were used to modify the scaffolds' porosity (75% and 85%). To enhance cell adhesion and interaction with porous PEEK, HA, CF, and CNTs were employed. When only 0.5 weight percent of CNTs were added to PEEK/HA, an improvement in yield strength of 43% (4.51 MPa) and an improvement in compression modulus of almost 186% (252.91 MPa) were seen. It was possible to create hierarchically tunable porous scaffolds from the nanoscale to the microscale with a mechanical strength similar to trabecular bone by utilizing a low-temperature 3D printing technique to create amorphous PEEK with carboxyl groups (PAEK-COOH). While the “carboxyl groups” can electrostatically stimulate HA mineralization, the PAEK-COOH scaffold's nanoporous surface helps to promote cell attachment. The scaffold offers a viable approach for the investigation of PEEK scaffolds in osteogenic applications since it offers superior osseointegration than PEEK without the need for extra active components [30].

Thermoplastic polymers, like “PEEK, PEKK, polyphenyl sulfone (PPSU), and polyethylene (PE)”, have smaller atomic numbers than titanium, which results in less streaks and halo antiquities in CT images while continuing tolerable mechanical stability and biocompatibility, increasing their use in oral and maxillofacial surgery extremely advantageous. Vacuum pressing, or pressing from granules or pellets, is one method of manufacturing PEEK in dentistry, while CAD/CAM (computer-aided design and computer-aided manufacturing) milling is another [29]. Prosthesis building, which can be done with subtractive or additive manufacturing methods, is the third step of the CAD/CAM system. Among these is additive manufacturing (AM), also referred to as 3D printing, which is a manufacturing process that uses jetting, sintering, melting, photocuring, and extrusion to build solid items layer by layer. The characteristics of the material can be further enhanced by postprocessing techniques including coating, polishing, and heat treatments after it has been created [18].

Cevik et al. (2023) did an in vitro study to check the utility of PEEK as a material for a framework for prostheses made up of maxillofacial silicone. According to the study's findings, PEEK might be used as a substitute substructure for silicone prostheses that are maintained in place by implants [32]. Luo et al. (2023) stated that the mechanical properties of 3D-printed PEEK can be influenced by printing temperature and speed, and it has superior flexural and tensile strength when compared to traditional pressed and CAD/CAM milling production methods [33]. Similar results were documented by Ding et al. in 2021 that PEEK composites that have been 3D printed have generally shown excellent tensile and flexural capabilities, showing them to have considerable potential for use in dental applications [34]. de Araújo Nobre et al. in 2023 studied 76 patients rehabilitated with all four concepts using PEEK as a material of choice, and they concluded after five years of follow-up that PEEK is regarded as a practical substitute for prosthetics [35].

## Conclusions

PEEK can be a great choice of material for prostheses and implants in the coming years. It has all the desired mechanical as well as physical properties, which are the same as that of bone. PEEK dental prosthesis tends to be good in managing masticatory forces. PEEK also has some disadvantages in its unmodified state, but surface modification enhances cell adhesion, proliferation, biocompatibility, and osteogenic properties of the material for successful dental implant therapy. Because of its advantageous mechanical, chemical, and physical qualities, PEEK has been recommended by a number of in vitro investigations and clinical reports as a potential material for permanent and removable dental prostheses made with CAD/CAM technology. Since there has been so little study on PEEK materials to date, it is still very early to claim that PEEK will eventually replace titanium implants. To confidently advocate PEEK as a substitute for proven prosthodontic materials, however, more non-clinical and clinical research is required to assess the prostheses for extended execution.
